# One-year outcome after laser treatment of vulvar lichen sclerosus: a prospective observational trial

**DOI:** 10.1007/s00404-026-08510-3

**Published:** 2026-07-01

**Authors:** Marianne Gamper, Irena Zivanovic, Helena Bischofberger, Volker Viereck

**Affiliations:** 1 Blasenzentrum der Frau AG, Lindenweg 7c, 8500 Frauenfeld, Switzerland; 2 Department of Gynecology and Obstetrics, Thurgau Hospital, Frauenfeld, Switzerland

**Keywords:** Neodymium:YAG laser, Erbium:YAG laser, RCT, 12-month follow-up, Crossover therapy, Corticosteroid

## Abstract

**Purpose:**

To assess the 12-month efficacy of a novel non-ablative dual Nd:YAG/Er:YAG laser therapy for vulvar lichen sclerosus (LS) and to evaluate the impact of crossover laser treatment following initial topical corticosteroid therapy.

**Methods:**

This study presents the 1-year data of a prospective observational trial. Between baseline and 6 months, women with vulvar LS were randomized to receive either dual Nd:YAG/Er:YAG laser therapy or topical clobetasol propionate. Between 6 and 12 months, optional steroid or laser therapies were offered.

**Results:**

In the laser group, 34 of 44 patients (77%) required no additional treatment between 6 and 12 months, indicating sustained treatment effects. The total LS score improved significantly during this period (mean change -0.35 ± 0.60; 95% CI -0.56 to -0.14; *p* = 0.004), while other parameters showed non-significant improvements. In the corticosteroid group, 20 of 22 patients (91%) opted for a crossover to laser therapy. Following three laser sessions, the total LS score improved markedly (mean change -1.50 ± 0.83; 95% CI -1.89 to -1.11; *p* < 0.001), primarily driven by a reduction in atrophy (*p* < 0.001). Patient-reported improvement (PGI-I) increased from 45 to 77% (*p* = 0.008). At the 12-month follow-up, outcomes were comparable between the two study groups.

**Conclusion:**

Non-ablative dual Nd:YAG/Er:YAG laser therapy resulted in significant and sustained clinical improvement in vulvar LS over 12 months. Crossover to laser therapy after initial corticosteroid treatment further enhanced both objective and subjective outcomes.

**Clinical trial identification number:**

NCT03926299; date of registration: 24 April 2019.

## What does this study add to the clinical work

**Table:** 

This study presents a novel non-invasive dual Nd:YAG/Er:YAG laser therapy for vulvar lichen sclerosus, showing sustained improvement up to 12 months. Furthermore, crossover laser treatment after first-line topical steroids further enhanced outcomes, offering a potential option for steroid-refractory patients.	

## Introduction

Lichen sclerosus (LS) is a chronic inflammatory skin disease that typically involves the anogenital area. It is associated with symptoms, such as severe itching and soreness, whitening of the skin, hyperkeratosis, atrophy, erosions, pain, dyspareunia, fissures, stenosis, agglutination, loss of the labia minora, or adhesions of the clitoral hood [1–3]. LS may have a high psychological burden with an impact on genital self-image, sexuality, mood disorders or depression, as well as quality of life [4]. Women affected with vulvar LS have a lifetime risk of 2–5% of developing squamous cell carcinoma [3, 5, 6].

Topical potent corticosteroid therapy was the first-line treatment for LS. It suppressed inflammation, reduced itching and pain symptoms, inhibited disease progression, and was recommended to be used long term [1]. Regular steroid applications following instruction was effective and safe [1]. Nonetheless, doctors and patients were looking for alternatives to steroids, also as an option for non-compliant patients or non-responders [3, 7]. Laser therapy, either with CO_2_ or Nd:YAG/(Er:YAG) lasers, significantly improved vulvar LS symptoms [7–10]. Most randomized-controlled comparisons of laser therapy with topical steroid showed significant improvements in both arms [7, 10–14]. Follow-ups were evaluated 1–6 months after the last intervention. Outcomes after a longer observation period were not yet available.

This prospective observational trial describes for the first time the 1-year outcome after laser treatment of LS. It is a follow-up of a randomized-controlled trial (RCT) comparing the dual Nd:YAG/Er:YAG laser therapy with high-potent topical steroid therapy [10, 15].

The analysis presented here pursued two objectives: First, to assess the effectiveness and safety of laser treatment over a period of up to 12 months for patients in the laser group, and second, to analyze the effect of crossover laser treatment between 6 and 12 months for patients in the steroid group.

## Methods

### Design

**Fig. 1:**
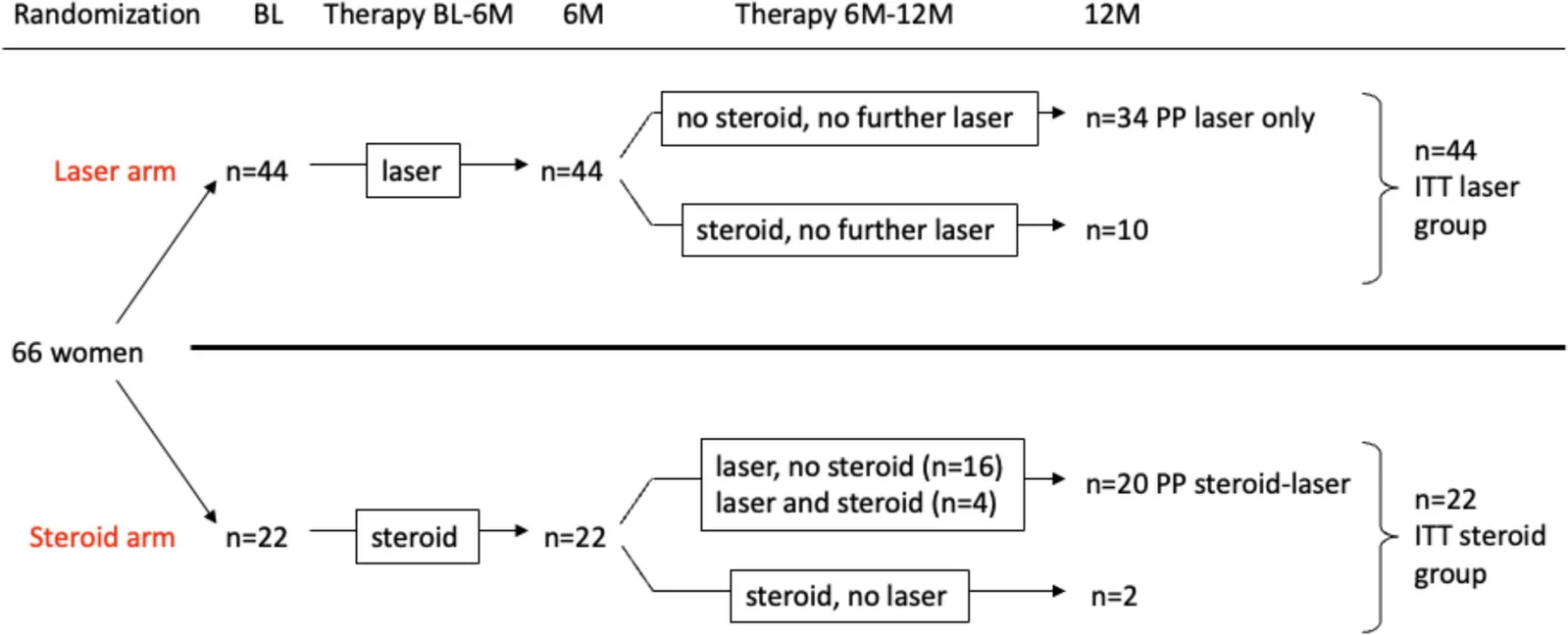
Flow diagram. After randomization at baseline and allocation to the laser (44 women) or steroid arm (22 women), outcome was evaluated at 6 and 12 months. Patients were free to choose their therapy between 6 and 12 months. Thirty-four women in the laser arm did not request a further treatment and were analyzed separately (“PP laser only”). Twenty women in the steroid arm requested crossover laser treatment and were analyzed separately (“PP steroid-laser”). The ITT analysis considered the original assignment to the study arms. There were no study dropouts. *BL* baseline, *6 M* 6-month visit, *12 M* 12-month visit, *PP* per-protocol, *ITT* intention-to-treat

This analysis investigated the 1-year prospective observational follow-up data of an investigator-initiated, single-center, active-controlled RCT carried out at an ambulatory tertiary referral center [10]. Baseline and 6-month data have been published [4, 10]. The study received ethical approval (EKOS 19/056, BASEC-ID: 2019-00634, 15 April 2019) and was registered (www.clinicaltrials.gov, NCT03926299, 24 April 2019). Patients gave informed consent. The study protocol [15] and the CONSORT diagram up to the 6-month follow-up [10] were published. The flow diagram between 6 and 12 months is shown in Fig. 1.

Women with a clinical diagnosis of vulvar LS and a physician-administered clinical LS score ≥ 4 were included [10, 16, 17]. The validated LS score was used in several clinical LS studies [3, 9, 18–20]. The total LS score assessed the six clinical parameters erosions, hyperkeratosis, fissures, agglutination, stenosis, and atrophy. Each parameter was scored 0 (normal/none), 1 (a few signs/moderate), or 2 (clear signs/severe) [3, 9, 16–18], yielding a total LS score from 0 to 12 (most severe). A total LS score of ≥ 4 identified LS with a probability of > 90% [16]. Further inclusion and exclusion criteria were previously described [10, 15].

### Study plan and intervention

Women in the laser arm received four laser treatments with the dual Nd:YAG/Er:YAG laser (FotonaSmooth SP^®^ Spectro laser, Model M021-4AF/3) within 4 months; women in the steroid arm received de-escalating doses of standardized topical clobetasol 0.05% for 6 months. No crossover treatment occurred between baseline and 6 months [10]. Laser settings and steroid dosage were previously published [10, 15].

At the 6-month visit, further treatment options were individually discussed with each patient. Between 6 and 12 months, patients could choose between no treatment, steroid treatment, and laser or laser/steroid treatment. Crossover laser treatment was free for women in the steroid arm, and complementary steroid maintenance therapy was recommended for women in both arms, especially younger women. Maintenance was defined by a continuous regular local steroid treatment once or twice a week, also in the absence of symptoms [21, 22]. For the optional crossover laser treatment in the steroid arm, laser settings and treatment regimen were the same as for the previous laser treatment in the laser arm [10, 15]. Three of the four laser sessions took place at 8, 9, and 10 months after baseline. The fourth laser session was right after the assessment of the study parameters at the 12-month visit.

### Outcomes and outcome measures

The primary outcome was the change of the objective clinical LS score in the laser arm between baseline and 6 months as previously described [10]. Secondary outcome measures were the changes of objective and subjective parameters after laser or steroid/laser therapy at 12 months [15]. Objective outcome was determined by the LS score [16, 17]. Subjective symptoms were evaluated by the vulvovaginal symptoms questionnaire (VSQ) [15, 23, 24], the visual analog scale (VAS) score [10], and the Patient Global Impression of Improvement questionnaire (PGI-I) [25]. VSQ questions 1–16 (symptoms, emotions, life impact) yielded a total VSQ score from 0 to 16 (most severe). The VAS score described the symptom strength on a scale from 0 to 10 (most severe). Itching, burning, and pain yielded a total VAS score from 0 to 30 (most severe). For PGI-I, women were asked to compare their vulvar condition at 6 and 12 months with their condition before the treatment. Treatment satisfaction was rated on a 7-point Likert scale, from −3 (very much worse) to + 3 (very much better) [10]. Pain during laser treatment was monitored with a VAS score from 0 to 10 (most severe).

### Statistical analysis

Wilcoxon signed-rank test (paired) was used to compare changes within groups and Wilcoxon rank sum/Mann–Whitney *U* test (non-paired) was used to compare changes between groups [Statistics Kingdom (https://www.statskingdom.com, accessed August 5 2025)]. Mean, standard deviations (SD), and confidence intervals (CI) were calculated with Excel. A p value below 0.05 was considered statistically significant; p values were rounded to 3 digits or indicated as < 0.001 where applicable. Data were calculated on an intention-to-treat (ITT) basis where the results were shown according to the assigned randomization arm, and on a per-protocol (PP) basis where the results were shown according to the received treatment.

## Results

Baseline and 6-month data including the primary outcome of this study have been published [4, 10]. Of the 66 study participants, 19 were premenopausal and 47 postmenopausal, while 74% (35/47) of the postmenopausal women used local estrogens. At baseline, the patients were randomized 2:1, 44 to the laser arm and 22 to the steroid arm. At 6 months, both laser and steroid therapies resulted in similar significant objective and subjective improvements (Table 1A). The between-group comparison showed significantly better outcomes after laser therapy for treatment satisfaction and for the clinical LS score including vulvar atrophy (Table 1B). After the 6-month visit, the women were free to choose their further treatment.

Between 6 and 12 months, none of the 44 women in the laser arm wanted a further laser treatment. Seventy-seven percent (34/44) were symptom-free and did not want steroid maintenance therapy. Their 12-month data, therefore, provided information on the long-term effectiveness of laser treatment (Fig. 1, “PP laser only”). Ten women (10/44) in the laser arm (23%) received steroid maintenance therapy. Seven of them started right after the 6-month visit because of itching (3x), a fissure (1x), or urinary incontinence (1x), or they were symptom-free and used steroid upon recommendation (2x). Three women started maintenance steroid therapy between 6 and 12 months due to aggravating itching symptoms (2x) or following a urinary tract infection (1x).

After the 6-month visit, 20 women in the steroid arm (20/22, 91%) wanted to participate in the crossover laser therapy, hoping to achieve further improvement following the partial improvement obtained with steroid therapy (Fig. 1, “PP steroid-laser”). Only four of them used accompanying steroids. Three continued with maintenance therapy—two upon medical recommendation and one due to severe atrophy—while the fourth patient began steroid treatment three months later because of aggravating itching symptoms. Two patients of the steroid arm (2/22, 9%) had no laser therapy and continued with steroid maintenance therapy, one was satisfied with steroid only and for the other one, laser therapy was postponed until after the 12-month visit due to COVID travel restrictions.

**Fig. 2:**
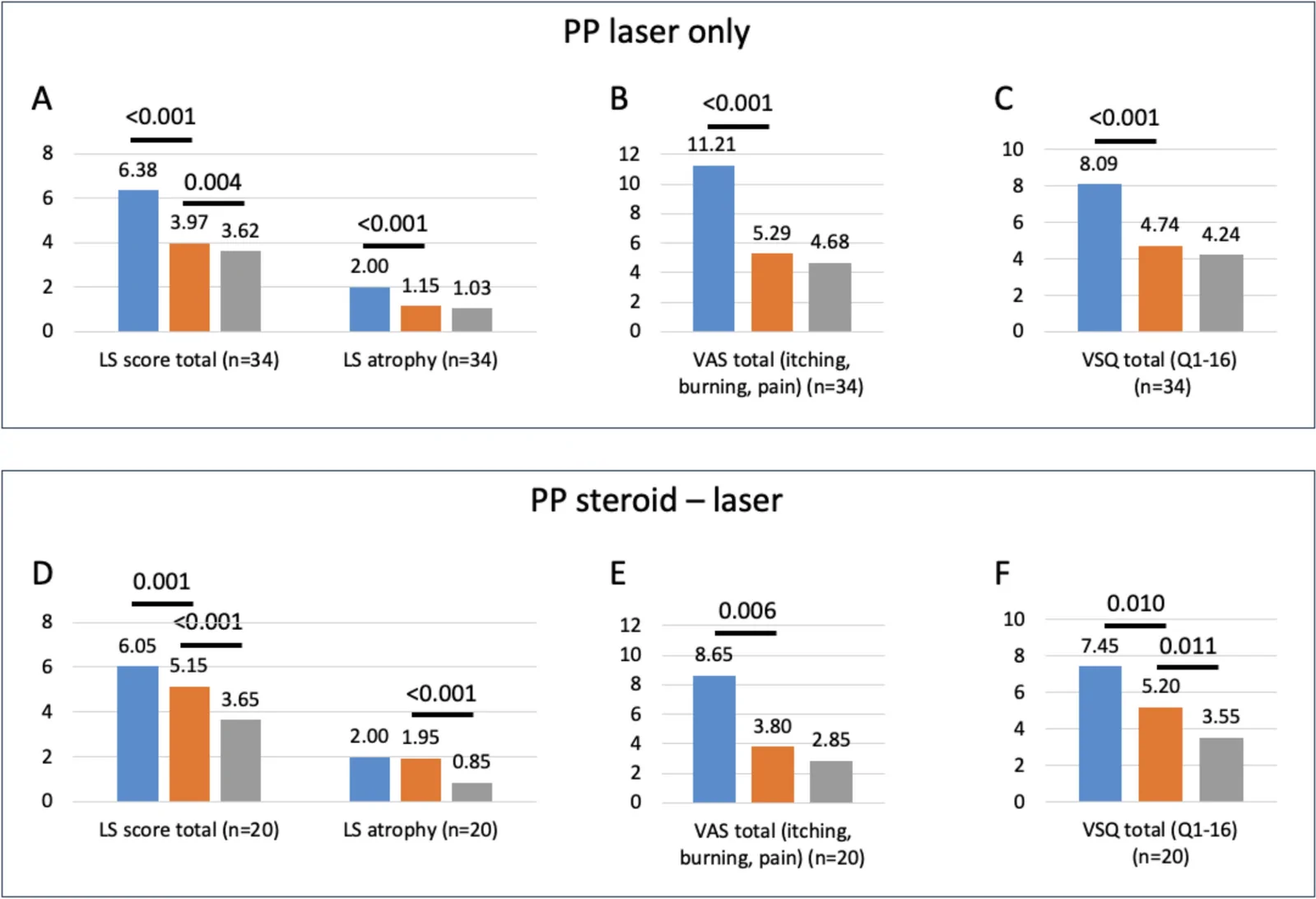
Per-protocol (*PP*) analysis. Baseline (blue), 6-month (orange), and 12-month (gray) data are shown. **A**, **B**, **C** Data of the 34 patients in the “PP laser only” group: the difference between blue and orange columns and that between orange and gray columns show the effect of four laser treatments between baseline and 4 months. **D**, **E**, **F** Data of the 20 patients in the “PP steroid-laser” group: The difference between blue and orange columns shows the effect of steroid treatment; the difference between orange and gray columns shows the effect of crossover laser treatment. **A**, **D** Total LS score (range 0–12) and LS atrophy (range 0–2); **B**, **E** total VAS score (itching, burning, pain) (range 0–30); **C** and **F** total VSQ score, questions 1–16 (range 0–16). Mean numbers and significant *p* values within are shown; see also Table 1A

For the 34 women in the laser arm who did not receive any further therapy (PP laser only), the initial four laser treatments were effective up to 12 months, i.e., up to 8 months after the last laser treatment. Notably, all investigated parameters improved between 6 and 12 months, non-significantly for total VAS (*p* = 0.629) and total VSQ (*p* = 0.325) (Fig. 2B, C, Table 1A), and significantly for the total LS score (*p* = 0.004; decrease −0.35 ± 0.60; 95% CI −0.56 to −0.14; Fig. 2A; Table 1A). Outcome numbers of all 44 women in the laser arm (ITT analysis) did not substantially differ from the PP data (Table 1A), indicating a generally good and stable outcome after laser therapy.

**Table 1:** Clinical LS score, VAS and VSQ at baseline, 6 and 12 months

A	Baseline mean ± SD	6 months mean ± SD	12 months mean ± SD	BL-6 M *p* value within	6 M–12 M *p* value within
**ITT laser group,** ***n*** **= 44**					
LS score total	6.27 ± 1.25	3.93 ± 1.34	3.61 ± 1.08	** < 0.001**	**0.024**
VAS total (itching, burning, pain)	11.59 ± 6.87	5.75 ± 6.50	4.39 ± 4.66	** < 0.001**	0.238
VSQ total (Q1–16)	8.11 ± 3.58	4.89 ± 4.42	4.23 ± 3.61	** < 0.001**	0.221
**ITT steroid group,** ***n*** **= 22**					
LS score total	6.05 ± 1.50	5.09 ± 1.15	3.68 ± 1.13	** < 0.001**	** < 0.001**
VAS total (itching, burning, pain)	9.36 ± 5.60	4.00 ± 3.12	2.68 ± 2.93	**0.002**	0.134
VSQ total (Q1–16)	7.68 ± 3.20	5.36 ± 4.27	3.77 ± 3.95	**0.005**	**0.013**
**PP laser only,** ***n*** **= 34**					
LS score total	6.38 ± 1.33	3.97 ± 1.24	3.62 ± 1.16	** < 0.001**	**0.004**
VAS total (itching, burning, pain)	11.21 ± 6.94	5.29 ± 6.10	4.68 ± 5.12	** < 0.001**	0.629
VSQ total (Q1–16)	8.09 ± 3.16	4.74 ± 4.29	4.24 ± 3.58	** < 0.001**	0.325
**PP steroid laser,** ***n*** **= 20**					
LS score total	6.05 ± 1.43	5.15 ± 1.18	3.65 ± 1.14	**0.001**	** < 0.001**
VAS total (itching, burning, pain)	8.65 ± 5.30	3.80 ± 2.55	2.85 ± 3.01	**0.006**	0.168
VSQ total (Q1–16)	7.45 ± 2.63	5.20 ± 3.83	3.55 ± 3.75	**0.010**	**0.011**

B	Baseline mean ± SD	6 months mean ± SD	12 months mean ± SD
**LS score total**			
ITT laser group, *n* = 44	6.27 ± 1.25	3.93 ± 1.34	3.61 ± 1.08
ITT steroid group, *n* = 22	6.05 ± 1.50	5.09 ± 1.15	3.68 ± 1.13
*p* value between	0.391	** < 0.001**	0.854
**VAS total (itching, burning, pain)**			
ITT laser group, *n* = 44	11.59 ± 6.87	5.75 ± 6.50	4.39 ± 4.66
ITT steroid group, *n* = 22	9.36 ± 5.60	4.00 ± 3.12	2.68 ± 2.93
*p* value between	0.304	0.647	0.203
**VSQ total (Q1–16)**			
ITT laser group, *n* = 44	8.11 ± 3.58	4.89 ± 4.42	4.23 ± 3.61
ITT steroid group, *n* = 22	7.68 ± 3.20	5.36 ± 4.27	3.77 ± 3.95
*p* value between	0.473	0.637	0.314

*ITT* intention-to-treat, *PP* per-protocol, *VSQ* vulvovaginal symptoms questionnaire, *Q1–16* specific questions of the VSQ, *VAS* visual analog scale, *LS score total* clinical lichen sclerosus score (including erosions, hyperkeratosis, fissures, agglutination, stenosis, atrophy), *BL* baseline, *6 M* 6 months, *12 M* 12 months. Statistically significant values are shown in bold

The 91% (20/22) of women in the steroid arm who received crossover laser treatment were analyzed separately (PP steroid laser). Crossover laser treatment between 6 and 12 months led to improvement of all investigated parameters (Fig. 2D–F; Table 1A). The total LS score improved significantly by −1.50 ± 0.83 (95% CI −1.89 to −1.11; *p* < 0.001) and the total VSQ score by −1.65 ± 3.00 (95% CI −3.05 to −0.25; *p* = 0.011), while the total VAS score improved non-significantly by −0.95 ± 3.71 (95% CI −2.69 to 0.79; *p* = 0.168). Improvement of the total LS score was due to a significant improvement of LS atrophy (*p* < 0.001, Fig. 2D) and LS hyperkeratosis (*p* = 0.020). Improvement of the total VSQ score was due to a significant improvement of VSQ symptoms (*p* = 0.030) and VSQ emotions (*p* = 0.034). Outcome data including all 22 women of the steroid arm (ITT analysis), i.e., also including the 2 women without crossover laser therapy, did not substantially differ from the PP data (Table 1A).

After 6 months, we previously reported a significant difference in the total LS score between the laser and steroid arms (ITT laser vs. ITT steroid: 3.93 ± 1.34 vs. 5.09 ± 1.15; *p* < 0.001 [10]; Table 1B). At 12 months, after 20 patients in the steroid arm had received crossover laser treatment, the mean total LS score of both arms was equally low (ITT laser vs. ITT steroid: 3.61 ± 1.08 vs. 3.68 ± 1.13; *p* = 0.854; Table 1B). Values of VAS total and VSQ total did not differ at any study visit between ITT laser and ITT steroid groups (Table 1B).

Patient satisfaction (PGI-I) with the overall treatment—based on an ITT analysis—improved in the steroid arm: 45% (10/22) felt very much better/much better at 6 months vs. 77% (17/22) at 12 months. The mean PGI-I value increased from 1.27 at 6 months to 1.95 at 12 months (*p* = 0.008). In the laser arm, PGI-I improved from 64% (28/44) at 6 months to 73% (32/44) at 12 months. The mean PGI-I value increased from 1.77 to 2.00 (*p* = 0.351). ITT laser vs. ITT steroid arm comparison at 6 months showed a higher satisfaction rate in the ITT laser (64%) vs. the ITT steroid arm (45%) (mean PGI-I values 1.77 vs. 1.27, *p* = 0.035) [10]. At 12 months, patients in both arms were equally satisfied (ITT laser 73% vs. ITT steroid 77%; mean PGI-I values 2.00 vs. 1.95, *p* = 0.936).

Nd:YAG/Er:YAG laser therapy was safe. No adverse event or malignancy was observed during the observation period of 12 months. Only one patient at one laser session indicated a minimal pain score of VAS 2 during laser treatment; all other 59/60 (20 × 3) laser treatments were painless (VAS 0).

## Discussion

Dual Nd:YAG/Er:YAG laser therapy resulted in significant improvements in both objective and subjective parameters of vulvar LS. The therapeutic effect persisted for up to 1 year, i.e., up to 8 months after the final laser treatment. Crossover laser therapy following prior steroid treatment further improved vulvar atrophy, hyperkeratosis, subjective symptoms, and treatment satisfaction. Overall, the laser procedure was well tolerated, safe, and painless.

The strength of this study lies in evaluating (1) the 1-year effectiveness of laser therapy and (2) the effect of laser therapy after steroid pre-treatment. Most patients in the laser arm (34/44) declined additional therapy between months 6 and 12, while the majority in the steroid arm (20/22) requested crossover laser treatment.

The study benefited from a high patient adherence without dropouts. The high compliance reflected the good tolerability of the treatments and a patient-friendly study design. From the beginning of the study, patients were informed that, if assigned to the steroid arm, they had the option to undergo a free crossover laser treatment. Most participants welcomed this option after the follow-up appointment at 6 months, even though the steroid treatment had already achieved partial relief. They were hoping to further improve their condition and quality of life; they were eager to test an alternative to long-term steroid therapy without side effects and wanted to extend the steroid-free interval. In addition, the personalized single-center setting, comprehensive documentation, and frequent follow-ups further supported treatment adherence.

This study has several limitations. The small sample size limits the statistical power and precludes subgroup analyses. Furthermore, external confounding factors cannot be ruled out during the 12-month follow-up period and should be considered when interpreting the results. Another limitation is that patients may already have a positive attitude toward the new laser technology before treatment begins. While well-informed patients use steroids correctly and generally benefit from them, some patients are skeptical of steroids and fear side effects with long-term use. In addition, three-to-four laser treatments may be more convenient than a regular application of topical corticosteroids. All these subjective attitudes could skew the study results in favor of laser therapy.

RCTs comparing CO₂ laser [11–13, 19, 20] or Nd:YAG laser therapy [7, 10] with steroid therapy [7, 10–13], sham treatment [19], or radiofrequency therapy [12] reported follow-up periods of only 1–6 months after the final intervention. While short-term follow-ups are appropriate for the initial evaluation of a new treatment modality, mid- and long-term observation is essential before clinical implementation.

This study presented the first 1-year follow-up outcome after laser therapy. No safety concerns were reported during the observation period. The four laser treatments between baseline and 6 months demonstrated stable and long-lasting subjective and objective effects. Interestingly, the outcome parameters even continued to improve from 6 to 12 months. This may be attributed to the slow, long-lasting, and laser-dose-dependent collagen and vascular remodeling process, initiated by laser-induced collagen contraction and followed by neo-collagenesis, neo-angiogenesis, collagen reorganization, and skin tightening [8, 26–29]. Neo-angiogenesis enhanced tissue microcirculation, thereby reducing degenerative and atrophic changes in the epithelium and promoting proper morphogenesis [28]. According to histological analysis, collagen fibers began to increase 3 weeks after laser treatment and collagen remodeling was observed for several weeks [26]. Repeated laser treatments (top-ups) prolonged the effect [30, 31]; and in our study, the effect may have lasted up to 12 months.

The 6-month data of this study showed that laser and steroid therapy improved subjective and objective outcome parameters similarly efficiently [10]. Significant differences between the two study arms were only found for the physician-administered clinical LS score and treatment satisfaction, both with better results in the laser group. The 12-month data showed that prior steroid treatment was not a contraindication for subsequent successful laser therapy, which agreed with the previous findings [11]. This crossover laser therapy further improved the results; hence, after 12 months, there were no more significant differences between the two study arms regarding the parameters examined (ITT analysis). Notably, the crossover laser group achieved these results after only three laser sessions, whereas the original laser group received four sessions. This suggests that three laser treatments may be sufficient to achieve clinical efficacy. However, a fourth session may further support collagen remodeling and neo-angiogenesis, potentially extending the duration of the therapeutic benefit. Additional research is required to determine the optimal number of laser sessions.

## Conclusion

Nd:YAG/Er:YAG laser therapy alleviated LS symptoms and improved the overall vulvar skin condition, with therapeutic effects persisting for at least one year. Evidence from a 4-year follow-up study on laser treatment of stress urinary incontinence indicated that the laser effect diminishes over time [32], and patients may seek retreatment as symptoms gradually recur. Therefore, longer follow-up is required to determine the durability of the therapeutic effect and to evaluate whether non-ablative laser therapy can be recommended as an alternative or adjunct to corticosteroids. No cases of malignancy were detected. Given the underlying cancer risk, close and continuous monitoring with vulvoscopy still remains essential [5].

## Data Availability

No datasets were generated or analyzed during the current study.
